# Optimizing polypharmacy management in older adults: impact of an interdisciplinary outpatient clinic on long-term function and quality of life

**DOI:** 10.1007/s41999-026-01442-w

**Published:** 2026-03-07

**Authors:** Victoria Roncal-Belzunce, Marta Gutiérrez-Valencia, Bernardo Abel Cedeño-Veloz, Ramón San Miguel Elcano, Virginia Ruiz Izquieta, Irene Guruceaga-Eguillor, Itxaso Marín-Epelde, Arkaitz Galbete, Marina Sánchez-Latorre, Maite Sarobe-Carricas, Nicolás Martínez-Velilla

**Affiliations:** 1 Institute of Smart Cities (ISC), Department of Statistics, Computer Science and Mathematics, Public University of Navarre (UPNA), Pamplona, Navarre Spain; 2 Navarrabiomed. Pamplona, Navarre, Spain; 3 Navarre Institute for Health Research (IdiSNA), Pamplona, Navarre Spain; 4 Innovation and Organization Unit, Navarre Health Service, Pamplona, Spain; 5 Department of Geriatrics, Hospital Universitario de Navarra (HUN), Irunlarrea 3, 31008 Pamplona, Navarre Spain; 6 Department of Pharmacy, Hospital Universitario de Navarra (HUN), Pamplona, Navarre Spain; 7 Faculty of Medicine, Universidad de Navarra, Pamplona, Spain

**Keywords:** Functional capacity, Older adults, Outpatient clinic, Polypharmacy, Quality of life

## Abstract

**Purpose:**

Polypharmacy is prevalent in older adults with chronic conditions, often leading to functional decline and diminished quality of life. The aim was to examine the effects of an interdisciplinary intervention on functional decline in community-dwelling older adults undergoing polypharmacy.

**Methods:**

This prospective observational study was conducted from June 2021 to May 2022 at a Spanish public hospital outpatient clinic. Participants were adults aged 75 and older, prescribed five or more medications. A multidisciplinary team performed comprehensive medication reviews alongside standard care. The primary outcome was the change in functional capacity, assessed using standardized scales.

**Results:**

Among 104 participants (mean age 86.2 years; 66% female), 376 recommendations were made, averaging 4.2 interventions per participant, with a 63.8% acceptance rate. No significant changes were found in the Barthel Index or Lawton scores. However, Short Physical Performance Battery (SPPB) scores increased by 1.1 points (95% CI 0.4–1.8) at six months. Quality of life improved at three months but not at 6 months, except for reduced anxiety and depression. Healthcare utilization decreased, shown by fewer primary care and emergency visits.

**Conclusion:**

Specialized polypharmacy management improved SPPB scores and short-term quality of life while reducing healthcare utilization, suggesting the importance of targeted interdisciplinary interventions in managing polypharmacy in older adults.

## Key summary points

**Aim:**

To evaluate the impact of an interdisciplinary outpatient clinic on functional decline and quality of life in older adults experiencing polypharmacy.

**Findings:**

The study found that specialized polypharmacy management improved Short Physical Performance Battery (SPPB) scores by an average of 1.1 points at 6 months and enhanced quality of life in the short term, particularly in reducing anxiety and depression. Healthcare utilization decreased, evidenced by fewer primary care and emergency visits.

**Message:**

Targeted interdisciplinary interventions could be essential for effectively managing polypharmacy and improving outcomes in older adults.

## Introduction

The aging population is expanding rapidly. As aging individuals often contend with multiple chronic health conditions, the prevalence of polypharmacy (use of five or more medications) has increased [1]. As a matter of fact, the World Health Organization (WHO) describes polypharmacy as a major global problem due to its prevalence and link to numerous negative consequences such as falls, frailty, disability, quality of life and mortality [1, 2].

Older adults are particularly susceptible to polypharmacy and adverse drug events due to specific characteristics that make them more vulnerable [3]. Polypharmacy leads to decreased physical function and lower levels of physical activity, which can result in increased healthcare utilization, decreased quality of life, threatened independence, and a higher risk of mortality [4–7]. Different plausible mechanistic hypotheses linking polypharmacy or inappropriate medications to physical performance have been proposed [8–10]. For instance, deprescribing potentially harmful medications—such as benzodiazepines, anticholinergics, and centrally acting analgesics—may enhance mobility by reducing sedative burden, improving attention and motor planning, and enhancing reaction time, gait stability and postural control. Additionally, adjustments to antihypertensive medications or vasodilators could improve orthostatic tolerance, thereby reducing dizziness or syncope and enhancing stability during ambulation.

It is essential for older adults to reside in environments that support and maintain their intrinsic capacity and functional ability in order to promote healthy aging [11]. The management of polypharmacy in older adults is a critical issue that affects not only individual health but also the sustainability of healthcare systems [7].

Given the complexity of their healthcare needs, older adults often require comprehensive assessments and multidisciplinary interventions [12]. In the context of polypharmacy, interdisciplinary initiatives are being proposed to address the needs of these patients [13–15]. However, many interventions have focused solely on medication-related variables, neglecting other essential aspects such as functional status or clinical variables [5, 16–18].

Previous studies have shown that interdisciplinary care involving both geriatricians and pharmacists can enhance the health outcomes of older adults undergoing polypharmacy. However, research on the specific influence of such interdisciplinary interventions on functional decline in older adults with polypharmacy is lacking [5, 16–18].

The primary aim of this study was to examine the effects of an interdisciplinary intervention on functional decline in older adults undergoing polypharmacy. We hypothesized that an interdisciplinary intervention focused on pharmacological optimization and collaborative decision-making would improve functional decline.

## Methods

### Design

This prospective observational study was conducted from June 2021 to May 2022 at an outpatient consultation with the Geriatrics Department of a Spanish public teaching hospital, HUN, located in Pamplona, Spain. The study protocol has been described elsewhere [19]. This study was approved by the Ethics Committee of the Navarra Government (EO 2021/16). Community-dwelling older adults experiencing polypharmacy received standard care from a multidisciplinary team consisting of a geriatrician, nurse, and clinical pharmacist.

### Sample and recruitment

Geriatricians identified patients referred for this specialized outpatient consultation from various sources, including primary care, other geriatric outpatient consultations, and patients discharged after hospitalization. Inclusion criteria were being at least 75 years old, having a life expectancy of at least three months, experiencing polypharmacy, and not being enrolled in medication-related clinical trials. Eligible patients were invited to participate in the study and written informed consent was obtained from those who agreed to participate.

### Procedure

**Fig. 1:**
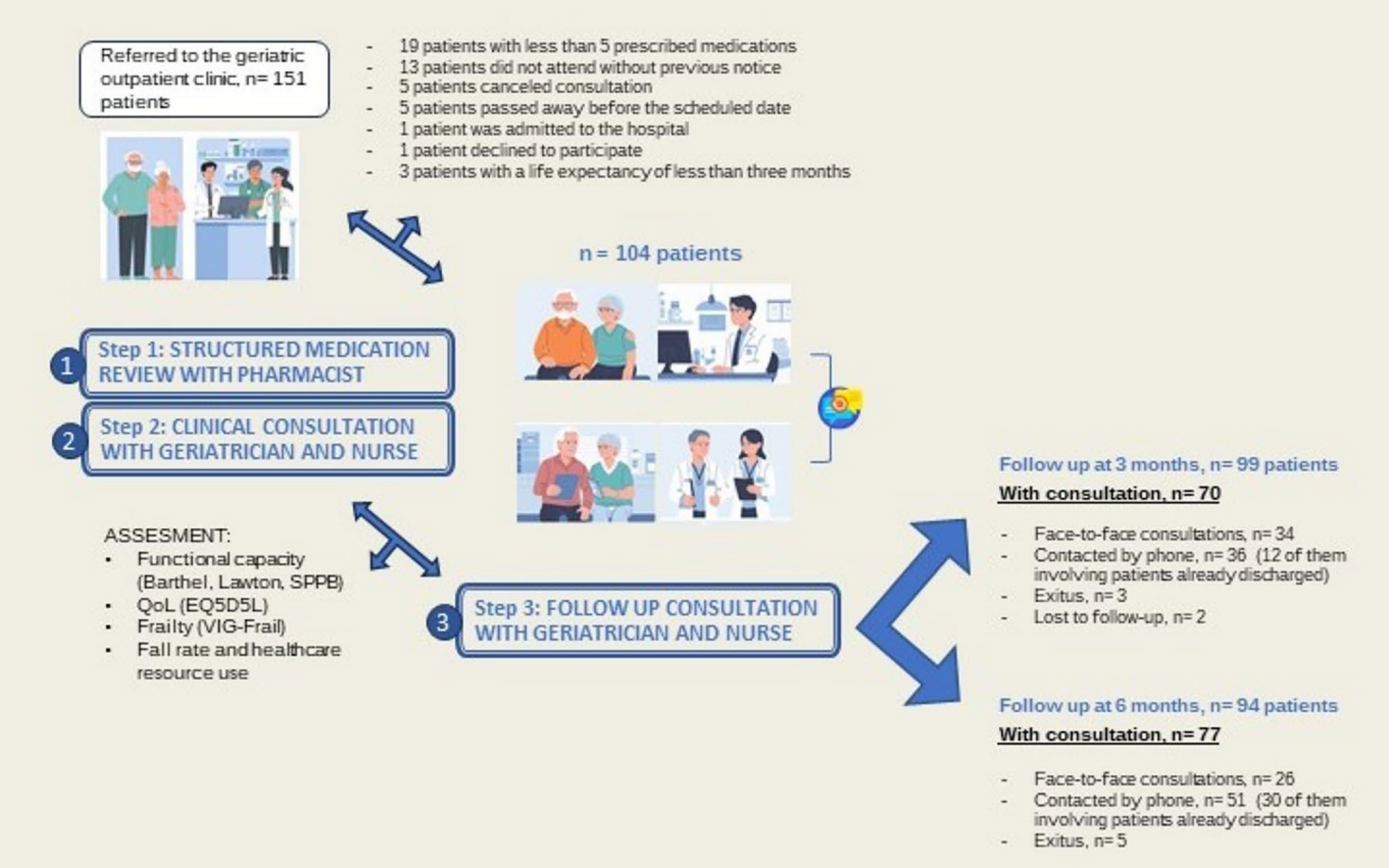
Study participant’s flow diagram

As part of the standard procedure, the clinical pharmacist routinely conducted a comprehensive medication review before each patient's initial visit (Fig. 1). The pharmacist developed medication recommendations based on the identified Drug-Related Problems (DRPs) and documented them in the patient's digital record, making them accessible to the geriatrician and nurse.

During the initial visit, the geriatrician and nurse performed a Comprehensive Geriatric Assessment (CGA), which included a medical interview with the patient or their caregiver, a physical examination, and verification of the current medication list with input from the patient and their family, among other activities. The pharmacist was not present at this visit. The geriatrician evaluated the appropriateness of the patient’s treatment regimen and reviewed the pharmacist’s recommendations. They then discussed these recommendations with the patient, considering their goals and preferences, and made the necessary medication changes in agreement with the patient.

The geriatrician, in collaboration with the nurse, scheduled follow-up consultations with the patient based on the patient's clinical status and treatment plan, which typically occurred approximately three and six months after the initial visit (in-person or via telephone). Both the geriatrician and the nurse were responsible for the long-term management of patients.

### Data collection

Data were collected at various stages: during the initial consultation (baseline period), and during follow-up assessments at 3 and 6 months. For patients discharged during the follow-up period, data were collected via the telephone. Patient characteristics and outcomes were documented during consultations and retrieved from the electronic medical records. All outcomes, except those related to healthcare utilisation, which were recorded from clinical records, were collected during follow-up only in those who had a follow-up consultation or telephone contact. The SPPB could only be collected in face-to-face consultations.

### Outcomes

The primary aim of this study was to evaluate changes in functional capacity, as assessed by the Barthel Index to assess basic ADLs (Spanish version; scale of 100 [functional independence] to 0 [severe functional dependence]) [20], Lawton scale to assess IADLs (Spanish version) [21], and Short Physical Performance Battery (SPPB) (total score ranging from 0 [worst] to 12 points [best]), which includes balance, gait, and rising from a chair test that can predict disability [22].

Secondary outcomes included changes in quality of life using the EQ-5D-5L questionnaire [23], frailty status determined by the Frail-VIG [24], rate of falling, and healthcare utilization metrics, such as hospital admissions, primary care visits, and emergency department visits. The EQ-5D-5L assesses self-perceived health status across five dimensions—mobility, self-care, daily activities, pain/discomfort, and anxiety/depression—with five response options: no problems, slight problems, moderate problems, severe problems, and extreme problems. The EQ-5D index or utility value was calculated using the Spanish EQ-5D-5L value set [25]. The proportion of patients with no problems, slight/moderate problems, and severe/extreme problems in each dimension was also calculated.

### Statistical analysis

Sociodemographic and clinical data were reported as frequencies and percentages for categorical variables, and as means and standard deviations for quantitative variables. Linear mixed models were used to compare quantitative variables from the baseline to various follow-up time points. The Wilcoxon signed-rank test was used to compare the EQ-5D dimensions, fall rates, and healthcare utilization. No imputation of missing data was performed. The significance level was set at *P* < 0.05. All statistical analyses were conducted using SPSS Statistics (version 28.0; IBM Corp., Armonk, NY, USA) and R software (version 4.3.2; R Foundation).

## Results

The study enrolled 104 patients during the study period, with an average age of 86.22 ± 5.32 years, and a demographic composition of 66% women. Figure 1 illustrates the patient flow throughout the study and Table 1 presents the characteristics of the enrolled patients.

**Table 1:** Characteristics of study participants

	Total (*n* = 104)
Comorbidity level	
CIRS-G mean (SD)	15.8 (5.0)
Cognitive function	
Without cognitive deterioration (GDS 1)	19 (18%)
Minor deterioration (GDS 2 and 3)	58 (56%)
Moderate deterioration (GDS 4 and 5)	20 (19%)
Major deterioration (GDS 6 and 7)	7 (7%)
Sensory deficiency	
Visual deficiency (including glasses and/or blindness)	81 (78%)
Hearing deficiency	76 (73%)
MNA	
Normal nutritional status (12–14)	32 (31%)
Risk of malnutrition (8–11)	45 (43%)
Malnutrition (0–7 points)	24 (23%)
Renal function, GFR (ml/min/1.73m2)	
≥ 60	61 (58.7%)
30–59	38 (36.5%)
15–29	2 (1.9%)
< 15	1 (1.0%)
Others:	
Total colesterol (mg/dL) mean (SD)	183.2 (47.2)
Total proteins (mg/dL) mean (SD)	65.1 (9.0)
Albumin (g/dL) mean (SD)	38.5 (5.7)
Hemoglobin (g/dL) mean (SD)	14.2 (12.6)
Fe (g/dL) mean (SD)	66.7 (31.6)
Creatine (g/dL) mean (SD)	0.99 (0.39)
Background	
Arterial hypertension	83 (80%)
Diabetes Mellitus	27 (26%)
Dyslipidemia	45 (43%)
Ischemic heart disease	21 (20%)
Heart failure	53 (51%)
Cardiac arrhythmia	37 (36%)
COPD/ asthma	20 (19%)
Depressive disorder	64 (62%)
Cerebrovascular pathology	52 (50%)
Chronic liver disease	5 (5%)
Chronic kidney disease	55 (53%)
Sleep disorder	76 (73%)
Marital status	
Single	11 (11%)
Married	42 (40%)
Divorced/ Separated	1 (1%)
Widower	50 (48%)
Educational level	
Without studies	7 (7%)
Primary education	55 (53%)
Secondary education, baccalaureate, or professional education	40 (38%)
University studies or equivalent	2 (2%)

CIRS-G, Cumulative Illness Rating Scale-Geriatric; COPD, chronic obstructive pulmonary disease; GFR, glomerular filtration rate; GDS, Global Deterioration Scale; MNA, Mini Nutritional Assessment

The results are presented as the mean (SD) or number (%)

During the study period, the pharmacist, as part of the multidisciplinary team, made 376 recommendations for 97 patients, covering 415 medications, averaging 3.6 recommendations per participant. Following the initial consultation, the geriatrician accepted 63.8% of these recommendations and, along with the multidisciplinary team, implemented 436 interventions, with an averaging 4.2 interventions per participant. Details about the medicines used and the changes that were made are described elsewhere [26].

### Primary outcome

#### Functional capacity

The Barthel Index and Lawton Index displayed no significant changes from baseline to the three- or six-month consultations. However, for the SPPB, although no statistically significant difference was observed at the 3-month consultation, a notable increase of 1.1 points (95% CI 0.4–1.8) was recorded at the 6-month assessment. Functional outcomes are presented in Table 2.

**Table 2:** Clinical and health care utilization outcomes

	Baseline	3 months consultation	Difference from baseline	*p*-value	6 months	Difference from baseline	*p-value*
Primary outcome							
Barthel	75.9 (21.7) n = 104	74.6 (23.1) n = 69	-0.2 (-2.3, 1.9)	0.852	75.8 (21.0) n = 72	-1.3 (-3.4, 0.7)	0.204
Lawton	3.0 (2.8) n = 104	2.6 (2.6) n = 67	-0.3 (-0.7, 0.1)	0.133	2.9 (2.9) n = 74	0.0 (-0.4, 0.4)	0.998
SPPB	5.6 (3.0) n = 94	7.1 (2.8) n = 26	0.3 (-0.3, 0.8)	0.342	7.4 (2.5) n = 15	1.1 (0.4, 1.8)	**0.006**
Secondary outcomes							
EQ-5D-5L Index	0.54 (0.24) N = 89	0.63 (0.15) N = 59	0.09 (0.03, 0.15)	**0.003**	0.58 (0.25) N = 59	0.04 (-0.02, 0.10)	0.238
Mobility							
Slight/moderate problems	56 (62.9%)	41 (69.5%)		**0.039**	40 (67.8%)		0.683
Severe/extreme problems	19 (21.3%)	7 (11.9%)			9 (15.3%)		
Self-care							
Slight moderate problems	51 (57.3%)	41 (69.5%)		0.429	30 (50.8%)		0.371
Severe/extreme problems	19 (21.3%)	7 (11.9%)			13 (22.0%)		
Usual activities							
Slight moderate problems	59 (66.3%)	43 (72.9%)		**0.008**	31 (52.5%)		0.827
Severe/extreme problems	22 (24.7%)	7 (11.9%)			17 (28.8%)		
Pain or discomfort							
Slight moderate problems	54 (60.7%)	44 (74.6%)		0.491	37 (62.7%)		0.933
Severe/extreme problems	14 (15.7%)	2 (3.4%)			5 (8.5%)		
Anxiety or depression							
Slight moderate problems	58 (65.2%)	39 (66.1%)		0.695	32 (54.2%)		**0.007**
Severe/extreme problems	14 (15.7%)	6 (10.2%)			7 (11.9%)		
EQ VAS	57.9 (16.6) *N* = 88	64.6 (13.0) *N* = 57	6.2 (1.7, 10.8)	**0.008**	62.8 (19.4) *N* = 58	4.2 (-0.4, 8.9)	0.074
Frailty Frail VIG	9.3 (3.4) *N* = 99	9.2 (3.6) *N* = 56	0.0 (-0.3, 0.4)	0.838	8.6 (3.2) *N* = 37	0.2 (-0.3, 0.6)	0.470

Values in bold are statistically significant

EQ-5D, Euroqol Quality of Life Scale for 5 domains; VAS, visual analogue scale; SPPB, Short Physical Performance Battery

### Secondary outcomes

#### Quality of life

**Fig. 2:**
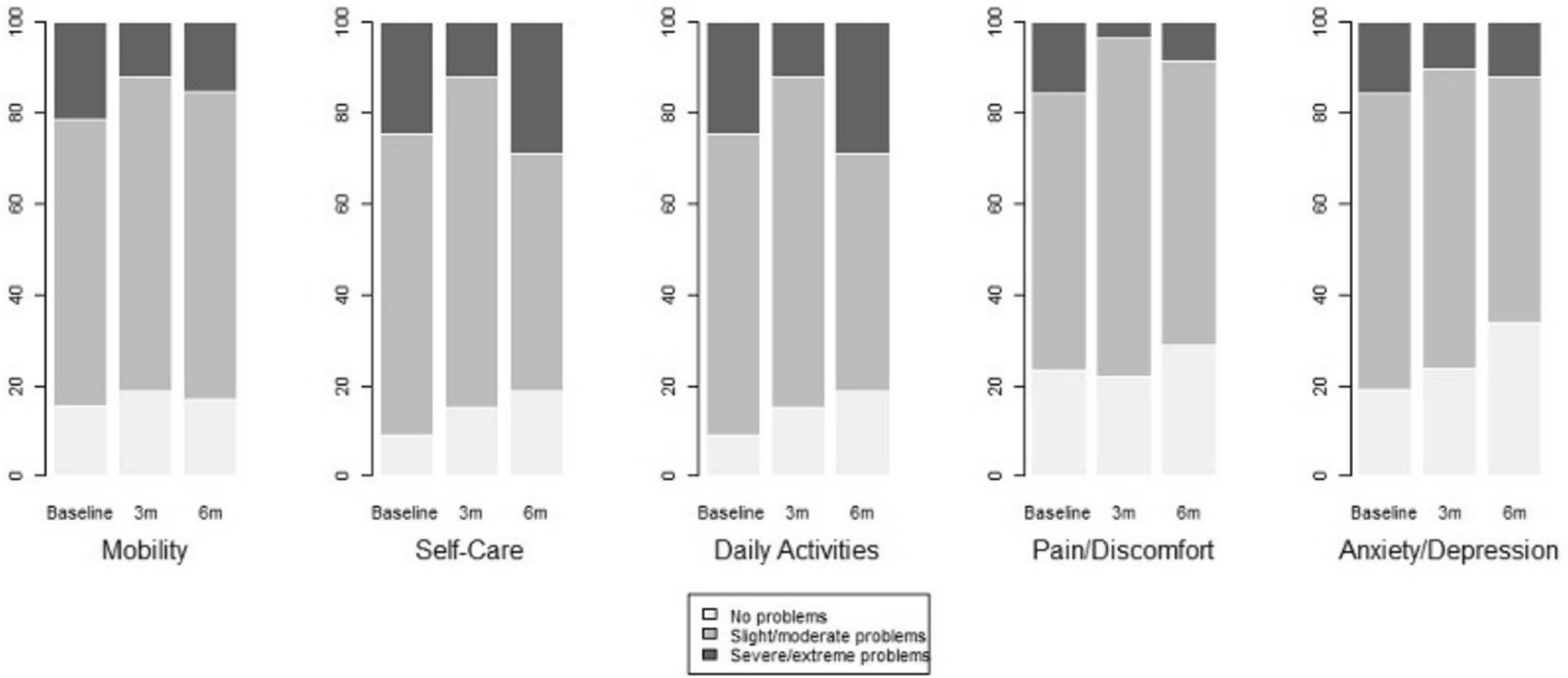
Proportion of patients in each category response for each EQ-5D-5L dimension in different time-points

At the three-month evaluation, both the EQ-5D-5L index and the Visual Analogue Scale exhibited a substantial increase, while no modifications from the baseline were observed at the six-month interval. Examination of each dimension of the EQ-5D-5L questionnaire individually revealed a decline in perceived difficulties related to mobility and daily activities at the three-month mark. Particularly, the proportion of patients experiencing severe or extreme mobility problems decreased from 21.3% to 11.9%, accompanied by a similar reduction in those experiencing severe or extreme difficulties in their usual activities. At the six-month interval, a statistically significant decrease in self-reported levels of anxiety or depression was noted (see Fig. 2).

#### Frailty

No significant variations were identified in frailty status throughout the follow-up period, as assessed by the VIG-Frail (Table 2).

#### Number of falls and healthcare resource use outcomes

Our findings revealed no differences in the occurrence of falls between the six months prior to the baseline consultation and the six months following it. However, we observed a minor increase in hospital admissions and a decrease in primary care and emergency visits during the latter period (Table 3).

**Table 3:** Number of falls and healthcare utilisation in the six months prior and after baseline consultation

	Previous 6 months	Subsequent 6 months	*p*-value
No of falls (*n* = 77)	1.2 (1.9)	0.8 (1.8)	0.165
No of hospitalizations (*n* = 94)	0.2 (0.5)	0.3 (0.6)	**0.015**
No of primary care visits (*n* = 94)	12.6 (7.5)	11.2 (8.7)	**0.011**
No of emergency visits (*n* = 94)	0.5 (0.8)	0.3 (0.6)	**0.017**

Values in bold are statistically significant

## Discussion

This investigation demonstrated that while there was no significant immediate improvement in Barthel and Lawton outcomes, the implementation of specialized, multidisciplinary polypharmacy management led to improved long-term SPPB and short-term quality of life, as well as a reduction in healthcare utilization. However, while our findings indicate initial improvements in quality of life and SPPB scores, the transient nature of these changes and the small, selected cohort could limit the generalizability of our results. Unlike prior studies, this study concentrates on the effects of polypharmacy interventions conducted by an interdisciplinary outpatient clinic on long-term outcomes.

Our findings align with a recent systematic review of 17 RCTs [27], which found mixed results for ADL and IADL improvements. However, the impact of interventions to address polypharmacy on long-term outcomes remains unclear. Our study cohort, representative of typical geriatric care settings often underrepresented in clinical trials, was predominantly older women with moderate comorbidity, varying cognitive function (mainly mild to moderate cognitive decline, GDS 2–5), and common visual and hearing impairments. Approximately two-thirds of the patients were at risk of malnutrition, consistent with previous findings that associated polypharmacy with malnutrition [28, 29]. Participants received an average of 3.6 recommendations, notably higher than in similar studies (2.6 to 3.2 per patient) [30, 31]. The 63.8% acceptance rate is generally considered favorable but slightly lower than that in studies with fewer recommendations (41.4–80.9%) [17, 30, 31], suggesting that the volume and complexity of recommendations may affect geriatricians’ acceptance.

Secondary outcomes revealed a significant improvement in quality of life at three months, especially in mobility and daily activities, but only anxiety and depression showed significant changes at six months. Short-term fluctuations in quality of life, which are common in older populations, may be influenced by the initial intervention, but tempered by time and existing comorbidities. A recent systematic review and meta-analysis of eight RCTs with similar interventions, populations, and settings revealed non-significant differences between the intervention and control groups with high heterogeneity and very low certainty [27]. Other systematic reviews to date have also shown heterogeneous results, with a predominance of studies that did not find a significant improvement in the quality of life [4, 14, 15, 32]. However, some authors have demonstrated significantly positive outcomes related to health-related quality of life [33].

No significant changes were observed in the post-intervention frailty or fall rates. However, there was a significant decrease in primary care and emergency department visits post-intervention, suggesting a potential influence on healthcare service utilization. The slight increase in hospitalizations could be a consequence of the detection of patients who are particularly susceptible to iatrogenic conditions and require an early approach. Further research is required to assess the impact of frailty on the prevention of falls. Although this has been suggested as a cautious strategy, previous studies have not conclusively demonstrated a clear improvement in frailty with reduced polypharmacy [34]. Intervention studies have yielded inconclusive results, necessitating further research to confirm potential benefits [35].

The existing literature lacks consensus on the relationship between multidisciplinary interventions for polypharmacy, falls, and hospital admission reduction. Several studies [36–38] found no significant differences in falls or hospitalizations. Although Lenander et al. observed some reduction [39], consistent evidence was lacking. Systematic reviews have presented mixed findings, with some studies showing no effect or insufficient evidence [14, 15, 40], whereas others have reported positive outcomes related to hospitalization reduction [33, 41]. Similarly, Roncal et al. (2024) found no significant differences in primary care or emergency department visits in their meta-analysis.

This study provides valuable insights into multidisciplinary interventions in geriatric care using a new consultation format. This pragmatic design ensures real-world applicability by maintaining standard practice and minimizing patient inconvenience. However, this study has some limitations, including a small sample size and a lack of diversity in the patient population, which may limit the generalizability of the findings. As this was not a randomized controlled trial (RCT), the absence of a control group and blinding limit our ability to draw causal conclusions and may introduce potential bias. In the absence of a control group, it is not possible to exclude the possibility of alternative explanations for the observed improvements. It is important to consider secular trends, whereby outcomes improve over time independently of the intervention. In addition, regression to the mean is particularly relevant in populations referred because of clinical instability, recent deterioration, or high symptom burden. On the other hand, regarding the SPPB, it is essential to consider that the sample size for measuring this outcome decreased from 94 to 26 at three months and 15 at six months, mainly due to limitations posed by telephone consultations. Potential selection bias due to attrition in SPPB assessments cannot be ruled out, as the loss of participants may skew the results toward those who are healthier and more robust. Therefore, this result must be interpreted with caution. Despite the limitations inherent in the study design, the results are favourable in terms of the feasibility, acceptability and early signs of clinical change of this type of intervention, which encourages further research in this area.

The study’s focus on routine clinical practice and the new consultation format meant that the pharmacist's involvement was limited to the initial consultation, without additional follow-up. Additionally, the six-month follow-up period might not have been long enough to detect significant changes in the quality of life and functioning, and the use of telephone consultations restricted our ability to monitor all variables over time. However, this allowed us to retain other data that would otherwise not have been captured. The low amount of data available for some variables at some points in the follow-up makes it necessary to interpret the results with caution.

## Conclusion

This study underscores the beneficial impact of specialized outpatient clinics on the management of polypharmacy among older patients. The observed short-term enhancements in quality of life and improvements in Short Physical Performance Battery (SPPB) scores, coupled with a reduction in healthcare utilization, suggest the necessity for ongoing evaluations and personalized interventions to maintain these benefits and guide healthcare policy development. Furthermore, it is imperative to pursue additional research aimed at elucidating the underlying mechanisms of polypharmacy and its clinical ramifications, as well as identifying biomarkers for the early detection of complications associated with polypharmacy. Such efforts will be crucial in optimizing care strategies for this vulnerable population.

## Data Availability

Data will be made available in reasonable request. Dr Martínez-Velilla (nicolasignacio.martinez@unavarra.es) should be contacted if someone wants to request the data from this study.
